# Adherence to monitoring of patients treated with amiodarone: a nationwide study

**DOI:** 10.3389/fmed.2024.1408799

**Published:** 2024-07-05

**Authors:** Amit Frenkel, Ruth Smadar Shneyour, Adi Shiloh, Mohammed Morad, Orly Shimoni-Rachmilev, Jacob Dreiher

**Affiliations:** ^1^General Intensive Care Unit, Soroka University Medical Center, Beer-Sheva, Israel; ^2^The Faculty of Health Sciences, Ben-Gurion University of the Negev, Beer-Sheva, Israel; ^3^Clinical Research Center, Soroka University Medical Center, Beer-Sheva, Israel; ^4^The Joyce and Irving Goldman Medical School, Faculty of Health Sciences, Ben-Gurion University of the Negev, Beer-Sheva, Israel; ^5^Risk Management and Patient Safety Unit, Kaplan Medical Center, Beer-Sheva, Israel; ^6^Pharmacy Services, Soroka University Medical Center, Beer-Sheva, Israel; ^7^Hospital Administration, Soroka University Medical Center, Beer-Sheva, Israel

**Keywords:** compliance, adherence, anti-arrhythmic drugs, monitoring, follow-up

## Abstract

**Objective:**

The aim of this study was to assess the adherence to monitoring guidelines regarding amiodarone treatment.

**Methods:**

This is a retrospective cohort study of data recorded in Clalit Health Services, the largest healthcare organization in Israel. Included were individuals aged >18 years; who were prescribed amiodarone and had a documented purchase of this drug, for a minimum of 200 consecutive days; and who had less than a 100-day gap between two consecutive purchases during 2013–2021. Adherence was assessed to testing for thyroid, liver function, and electrolytes, as determined by the performance of a test every 6 months.

**Results:**

The study included 24,094 individuals (mean age: 75 years, 53% male). The median follow-up was 2.3 years (total 73,727 person-years). The proportions of patients who performed baseline tests were: 43.4% for thyroid function, 58.3% for electrolytes, 48.6% for liver function, 20.6% for chest X-rays, and 14.9% for electrocardiograms. Adherence rates to semiannual monitoring of thyroid function, liver function, and electrolyte tests were: 70.4%, 79.4%, and 88.3%, respectively. In a multivariable analysis, the factors associated with higher adherence were male sex; older age; the presence of thyroid abnormalities, renal failure, and hypertension; and more frequent visits to the primary care physician.

**Conclusions:**

In our country, adherence is low to monitoring risk factors for adverse effects of amiodarone therapy, especially prior to treatment initiation. Patient and primary care physicians should be educated about the importance of monitoring, particularly prior to initiation of amiodarone treatment.

## Introduction

Amiodarone, a noncompetitive antagonist of alpha- and beta-adrenergic receptors and a type III antiarrhythmic agent, is frequently prescribed for ventricular and supraventricular arrhythmias. Despite its efficacy, challenges to amiodarone use in clinical practice include its prolonged half-life, multiple adverse effects, and drug interactions. These adverse effects are particularly problematic for older people who are more susceptible to drug toxicities (1). Within the first year of use, up to 15% of patients may experience adverse effects, with long-term use increasing the risk to 50% (2). Corneal microdeposits are the most common side effect, occurring in at least 90% of patients. Amiodarone may also prolong the QRS duration and QTc interval, leading to cardiovascular complications such as bradycardia, hypotension, Torsades de pointes, and conduction abnormalities (3). Pulmonary toxicity is a well-described side effect that often resembles interstitial lung disease, and that has been reported as closely related to the total cumulative amiodarone dose (4). This side effect may also manifest as organizing pneumonia, pleural effusion, acute respiratory distress syndrome, or diffuse alveolar hemorrhage. It can present at any time during treatment, though it typically presents in the first year (4). Prevalences of amiodarone-induced thyroid function abnormalities have been reported in the range of 4%−30% for hypothyroidism and 5%−6% for hyperthyroidism, depending on maintenance doses (5). The annual incidence of liver toxicity was reported as 1% (6). Although this condition mostly resolves after drug discontinuation, some patients may progress to end-stage liver disease and cirrhosis (6). Given the potential for adverse effects and the long half-life of amiodarone, early detection of initial damage is crucial.

Surprisingly, despite the well-known side effects and widespread use of amiodarone, only a few guidelines have been issued regarding monitoring patients who undergo outpatient treatment with this drug (7–9). Review of the main published guidelines shows general agreement that the necessary tests can be divided into three groups. First, baseline tests are recommended at the start of treatment, including liver function tests, thyroid function tests, a chest X-ray, and an electrocardiogram (ECG). Some guidelines also suggest ophthalmologic evaluations and pulmonary function tests. Second, routine tests should be performed at fixed intervals, either yearly or every 6 months. These include liver and thyroid function tests, and chest X-rays. Third, certain tests are recommended following the appearance of symptoms that may indicate a side effect of the medication. Examples of such are ophthalmologic evaluations and pulmonary function tests, high-resolution computerized tomography scans, and ECGs.

The primary goal of this study was to evaluate, in a large population, the rates of performing the recommended tests prior to treatment with amiodarone, and at 6-month intervals during the treatment.

## Methods

This is a retrospective cohort based on the computerized database of Clalit Health Services (Clalit), the largest healthcare provider organization in Israel. Clalit provides health care to more than 4.5 million persons, and operates more than 1,500 primary care clinics. Data were extracted from the Clalit Data sharing platform powered by MDClone (https://www.mdclone.com). This platform contains both demographic and clinical data for the patient's lifespan.

The patients were Clalit enrollees older than 18 years who who were prescribed amiodarone for any indication, for a minimum of 200 consecutive days, and who had less than a 100-day gap between two consecutive purchases during 2013–2021. The follow-up was limited to the period of time during which each patient was taking amiodarone regularly, as defined above, during 2013–2021. Patients were excluded from the analysis if they had missing medical or demographic data, were not Israeli citizens, and left Clalit prior to January 1, 2014.

For each patient, the data included demographic information, such as age and sex; clinical characteristics, such as chronic medications, chronic co-morbidities, information on adherence parameters, the number of clinic visits, and the results of laboratory tests, imaging studies, ECG, and imaging studies.

Baseline test parameters were defined as tests that were conducted within a time window extending from up to 30 days before and after the initial purchase of amiodarone. For follow-up parameters, we calculated the mean number of tests per year for each patient, based on their individual follow-up time. This calculation was done by dividing the total number of tests by the number of follow-up years for each patient. Patients were considered adherent to follow up tests if they had a test every 6 months. For example, a patient who was given amiodarone for 2 years would have to perform four tests to be considered adherent.

### Statistical analysis

Descriptive statistics, such as means and standard deviations, were calculated for all the variables in the analysis. In a univariable analysis, we compared characteristics between patients who were and were not adherent to each test performed at baseline and at 6-month intervals after treatment initiation. Stratified analysis for adherence to baseline and follow-up tests according to age and sex was done.

Logistic regression multivariable models were used to evaluate associations of baseline and follow-up tests for each type of test, separately, and independent factors such as demographics and clinical variables; with outcomes of non-adherence. A two-sided 5% significance test was considered as the maximal significance level in testing the study hypotheses. Statistical analysis was conducted using R software (https://www.r-project.org/). The study was approved by the institutional review board of Soroka Medical Center, in accordance with the principles of the Helsinki declaration (June 2022/0096-22-SOR).

## Results

A total of 24,094 patients were included in this retrospective study (mean age: 74.7 years, 53.2% male). Demographic and clinical characteristics of the study population are presented in Table 1. The median follow-up was 2.3 years (total 73,727 person-years). Complications of amiodarone therapy included hypothyroidism in 23.1%, thyrotoxicosis in 2.4%, liver cirrhosis in 1.7%, and peripheral neuropathy in 5.8% (Table 1).

**Table 1:** Demographic and clinical characteristics of the study population, *N* = 24,094.

**Variables**	**Values**
**Age, years**	
Mean	74.7 ± 10.8
Median	76
Range	18–104
Male sex (%)	12,830 (53.2)
**Amiodarone daily dose (%)**	
<200 mg	4,982 (20.7)
200–400 mg	18,274 (75.8)
>400 mg	837 (3.5)
**Underlying diagnoses (%)**	
Hypothyroidism	6,064 (25.2)
Thyrotoxicosis	957 (4.0)
**Complication of amiodarone therapy (%)**	
Hypothyroidism	5,563 (23.1)
Thyrotoxicosis	573 (2.4)
Liver cirrhosis	410 (1.7)
Peripheral neuropathy	1,405 (5.8)

The proportions of patients who performed baseline tests were: 43.4% for thyroid function tests, 58.3% for electrolytes, 48.6% for liver function, 20.6% for chest X-rays, and 14.9% for ECGs. The proportions of patients who adhered to semiannual monitoring of thyroid function, liver function, and electrolytes were 70.4%, 79.4%, and 88.3%, respectively. The proportions that completed all the recommended baseline and follow-up tests were 3.1% and 63.6%, respectively (Table 2). We estimated adherence to baseline tests for people without underlying comorbidity relevant to the test in question. Adherence rate to baseline thyroid function testing decreased to 29.1% when excluding patients with underlying thyroid disease. Similarly, adherence to electrolytes testing decreased to 45.9% in patients without underlying renal disease, and chest X-rays where done in 14.4% for patients without asthma or COPD. Of note, adherence to liver function tests, at 48.0% was nearly identical when excluding patients with liver disease.

**Table 2:** Adherence to baseline and follow-up studies.

**Variables**	**Values *N* = 24,094**
**Baseline monitoring (%)**	
Thyroid function tests	10,468 (43.4)
Electrolytes	14,050 (58.3)
Liver function tests	11,712 (48.6)
Chest X-ray	4,965 (20.6)
Electrocardiogram	3,584 (14.9)
All required studies	742 (3.1)
**Follow-up studies every 6 months**	
Thyroid function tests	16,969 (70.4)
Electrolytes	21,282 (88.3)
Liver function tests	19,039 (79.0)
All required studies	15,332 (63.6)
**Mean annual frequnecy** ^*^	
Primary care physician visits	5.79 ± 7.86
Ophthalmology clinic visits	2.09 ± 4.11
Pulmonary clinic visits	0.36 ± 1.45
Pulmonary function tests	0.12 ± 0.52
Thyroid function tests	4.99 ± 6.11
Electrolytes	12.58 ± 18.66
Creatine kinase	2.94 ± 5.53
Liver function tests	8.88 ± 13.38
Chest X-ray studies	2.06 ± 4.26
Electorcardiogram	1.22 ± 4.26

^*^The data is presented as mean ± standard deviation.

In a sensitivity analysis, we examined adherence to annual, rather than semiannual, testing. Adherence rates to annual monitoring of thyroid function, liver function, and electrolytes were 89.2%, 92.3%, and 96.5%, respectively. The proportion that completed all the recommended annual follow-up tests was 84.7%.

A stratified analysis by age and sex of adherence rates for baseline and follow-up test are shown in Table 3 Follow up test for thyroid functions tests were done for a higher proportion among females. Adherence increased with increasing age for baseline and follow-up electrolytes and liver function tests (Table 3). No other consistent differences were found.

**Table 3:** Adherence to baseline and follow-up studies, stratified analysis by sex and age.

**Strata by gender**			
**Characteristics**	**Female** ***N*** = **11,264**	**Male** ***N*** = **12,830**	***p*** **-value**
**Baseline monitoring**			
Thyroid function tests	5,032 (44.7)	5,436 (42.2)	<0.001
Electrolytes	6,419 (57.0)	7,831 (59.5)	<0.001
Liver enzymes Baseline (%)	5,308 (47.1)	6,404 (49.9)	<0.001
Chest X-ray	2,197 (19.5)	2,768 (21.6)	<0.001
All required studies (%)	342 (3.0)	400 (3.1)	0.743
**Follow-up studies every 6 months**			
Thyroid function tests	8,274 (73.5)	8,695 (67.8)	<0.001
Electrolytes	9,934 (88.2)	11,348 (88.4)	0.549
Liver enzymes Baseline (%)	8,856 (78.6)	10,183 (79.4)	0.16
All required studies (%)	3,871 (34.4)	4,891 (38.1)	<0.001
**Strata by age**						
**Characteristics**	**18–30** ***N*** = **39**	**31–50** ***N*** = **479**	**51–70** ***N*** = **6,927**	**71–80** ***N*** = **8,240**	>**80** ***N*** = **8,409**	***p*** **for trend**
**Baseline monitoring**						
Thyroid function tests	16 (41.0)	209 (43.6)	2,988 (43.1)	3,613 (43.8)	3,642 (43.3)	0.914
Electrolytes	14 (35.9)	264 (55.1)	4,052 (58.5)	4,873 (59.1)	4,847 (57.6)	0.007
Liver enzymes Baseline (%)	16 (41.0)	230 (48.8)	3,578 (50.2)	4,037 (49.0)	3,951 (47.0)	0.002
Chest X-ray	7 (17.9)	121 (25.3)	1,588 (22.9)	1,763 (21.4)	1,486 (17.7)	<0.001
All required studies (%)	2 (5.1)	23 (4.8)	242 (3.5)	252 (3.1)	223 (2.7)	0.006
**Follow-up studies every 6 months**						
Thyroid function tests	24 (61.5)	321 (67.0)	4,836 (69.8)	5,977 (72.5)	5,811 (69.1)	<0.001
Electrolytes	29 (74.4)	386 (80.6)	6,068 (87.6)	7,467 (90.6)	7,332 (87.2)	<0.001
Liver enzymes baseline (%)	27 (69.2)	355 (74.1)	5,495 (79.3)	6,685 (81.1)	6,477 (77.0)	<0.001
All required studies (%)	17 (43.6)	193 (40.3)	2,550 (36.8)	2,817 (34.2)	3,185 (37.9)	<0.001

**Figure 1:**
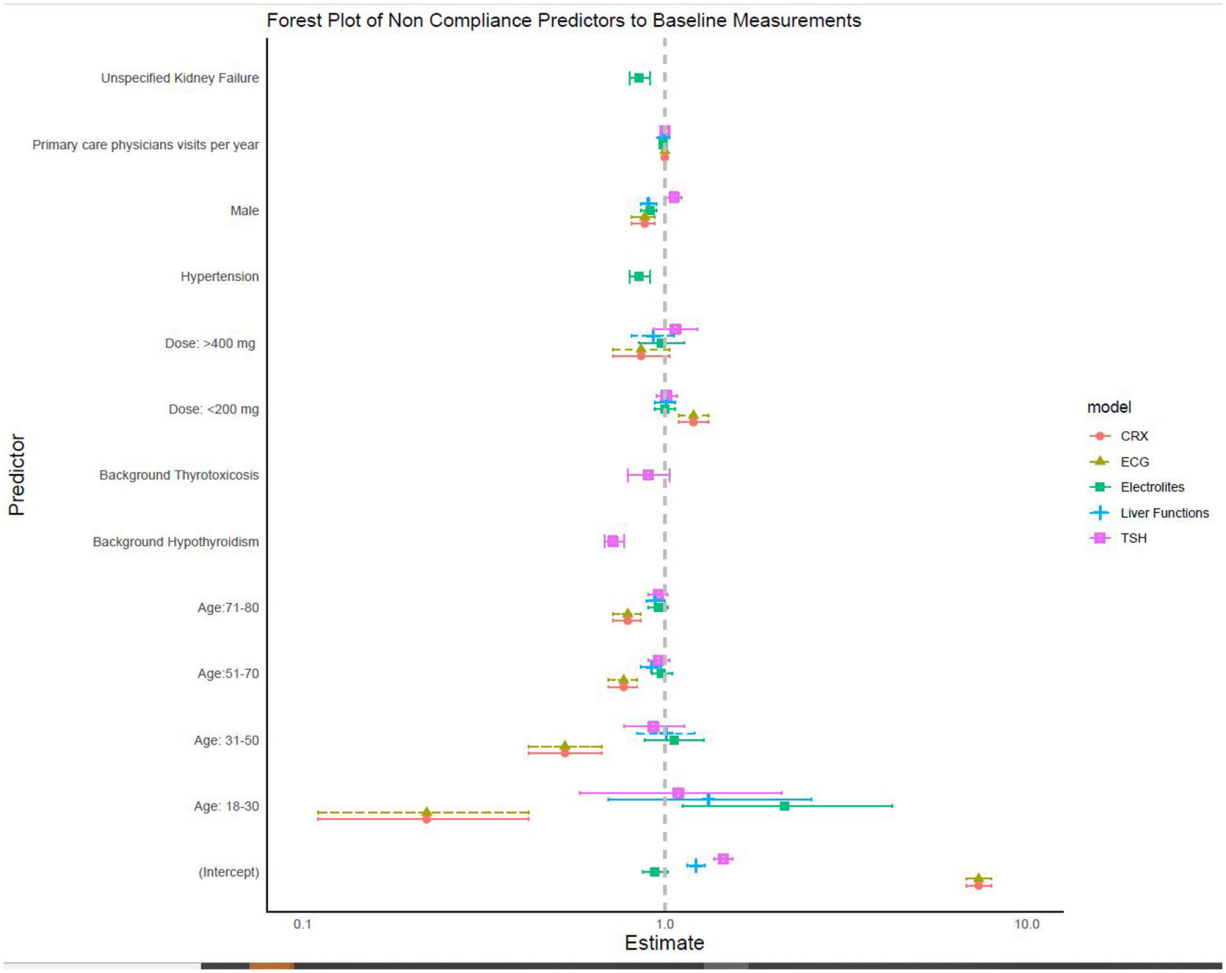
Factors associated with non-adherence to baseline studies (multivariable logistic regression models, *N* = 24,094).

In a multivariable analysis, the factors that were associated with performance of baseline thyroid function tests were female sex, more frequent visits to the primary care physician, and underlying hypothyroidism. The factors that were associated with performance of baseline liver function tests were younger age, male sex, and more frequent visits to primary care physicians. Performances of baseline chest X-rays and ECGs were more likely in younger patients and males, while a daily amiodarone dose <200 mg was associated with not performing these two tests (Figure 1 and Table 4).

**Table 4:** Adherence to baseline and follow-up studies, multivariable analysis, logistic regression models.

**Non adherence to baseline**														
**Characteristic**	**TSH**			**Liver Enzymes**			**Electrolytes**			**CXR**			**ECG**		
	**OR**	**95% CI**	***p*** **-value**	**OR**	**95% CI**	***p*** **-value**	**OR**	**95% CI**	***p*** **-value**	**OR**	**95% CI**	***p*** **-value**	**OR**	**95% CI**	***p*** **-value**
**Age**														
>80	—	—		—	—		—	—		—	—		—	—	
18–30	1.51	0.74, 3.00	0.2	1.70	0.81, 3.43	0.15	1.99	0.88, 4.20	0.083	1.07	0.50, 2.64	0.9	0.22	0.11, 0.42	< 0.001
31–50	1.33	1.07, 1.64	0.010	1.70	1.35, 2.13	< 0.001	2.04	1.57, 2.64	< 0.001	0.68	0.55, 0.85	< 0.001	0.53	0.42, 0.67	< 0.001
51–70	1.12	1.03, 1.20	0.005	1.18	1.08, 1.28	< 0.001	1.28	1.15, 1.42	< 0.001	0.77	0.71, 0.83	< 0.001	0.77	0.70, 0.84	< 0.001
71–80	1.00	0.93, 1.07	>0.9	0.99	0.91, 1.07	0.8	0.92	0.83, 1.02	0.12	0.82	0.76, 0.88	< 0.001	0.79	0.72, 0.86	< 0.001
**Gender**														
Female	—	—		—	—		—	—		—	—		—	—	
Male	1.23	1.16, 1.31	< 0.001	0.93	0.87, 1.00	0.043	0.94	0.86, 1.02	0.15	0.92	0.86, 0.98	0.012	0.88	0.81, 0.94	< 0.001
**Dosage (mg)**														
200–400	—	—		—	—		—	—		—	—		—	—	
Above 400	0.83	0.70, 0.98	0.032	0.83	0.68, 1.00	0.055	0.81	0.63, 1.03	0.089	0.88	0.75, 1.04	0.13	0.86	0.72, 1.03	0.094
Below 200	0.77	0.72, 0.83	< 0.001	0.70	0.64, 0.76	< 0.001	0.58	0.52, 0.65	< 0.001	1.38	1.27, 1.51	< 0.001	1.20	1.09, 1.32	< 0.001
**Additional factors**														
Background thyrotoxicosis	0.92	0.78, 1.07	0.3												
Background hypothyroidism	0.46	0.42, 0.49	< 0.001												
Primary care physician visits per year	0.81	0.80, 0.82	< 0.001	0.79	0.78, 0.80	< 0.001	0.71	0.70, 0.73	< 0.001	1.00	1.00, 1.00	0.8	1.00	0.99, 1.00	0.043
Unspecified kidney failure							0.75	0.67, 0.84	< 0.001						
Chronic hypertension							0.66	0.60, 0.73	< 0.001						
COPD										1.03	0.95, 1.12	0.4			
Asthma										0.94	0.85, 1.04	0.2			
**Characteristic**	**TSH**			**Liver enzymes**			**Electrolytes**		
	**OR**	**95% CI**	***p*** **-value**	**OR**	**95% CI**	***p*** **-value**	**OR**	**95% CI**	***p*** **-value**
**Age**									
>80	—	—		—	—		—	—	
18–30	1.51	0.74, 3.00	0.2	1.70	0.81, 3.43	0.15	1.99	0.88, 4.20	0.083
31–50	1.33	1.07, 1.64	0.010	1.70	1.35, 2.13	< 0.001	2.04	1.57, 2.64	< 0.001
51–70	1.12	1.03, 1.20	0.005	1.18	1.08, 1.28	< 0.001	1.28	1.15, 1.42	< 0.001
71–80	1.00	0.93, 1.07	>0.9	0.99	0.91, 1.07	0.8	0.92	0.83, 1.02	0.12
**Gender**									
Female	—	—		—	—		—	—	
Male	1.23	1.16, 1.31	< 0.001	0.93	0.87, 1.00	0.043	0.94	0.86, 1.02	0.15
**Dosage (mg)**									
200–400	—	—		—	—		—	—	
>400	0.83	0.70, 0.98	0.032	0.83	0.68, 1.00	0.055	0.81	0.63, 1.03	0.089
< 200	0.77	0.72, 0.83	< 0.001	0.70	0.64, 0.76	< 0.001	0.58	0.52, 0.65	< 0.001
**Additional factors**									
Background thyrotoxicosis	0.92	0.78, 1.07	0.3						
Background hypothyroidism	0.46	0.42, 0.49	< 0.001						
Primary care physician visits per year	0.81	0.80, 0.82	< 0.001	0.79	0.78, 0.80	< 0.001	0.71	0.70, 0.73	< 0.001
Unspecified kidney failure							0.75	0.67, 0.84	< 0.001
Chronic hypertension							0.66	0.60, 0.73	< 0.001

OR, odds ratio; CI, confidence interval.

**Figure 2:**
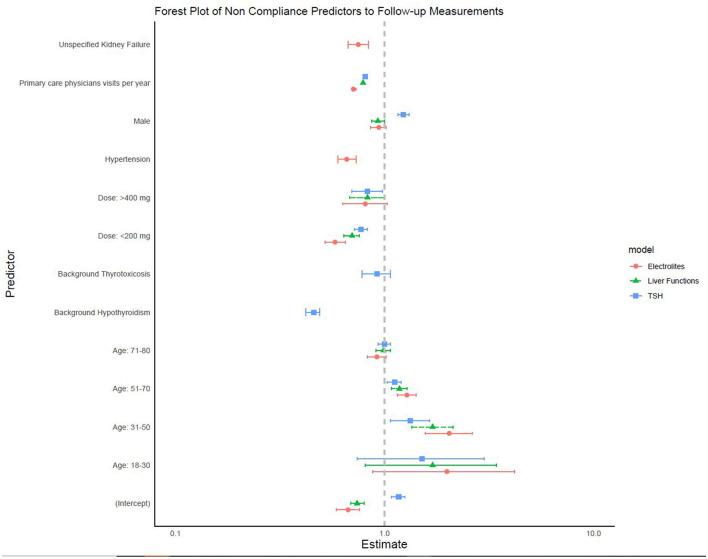
Factors associated with semi-annual follow-up studies.

Finally, a multivariable analysis showed that factors associated with adherence to semiannual thyroid function tests were older age, female sex, more frequent visits to primary care physicians, and either a low or a high daily dose of amiodarone. Factors that were associated with higher adherence to regular liver function test monitoring were older age, male sex, low daily amiodarone dose, and more frequent visits to primary care physicians. Performing regular electrolyte tests were more likely in older patients and males, patients on a daily amiodarone dose <200 mg, with underlying hypertension and chronic renal failure, and who more frequently visited primary care physicians (Figure 2 and Table 4).

## Discussion

In this large, population-based study of 24,094 patients treated with amiodarone during a period of over 8 years in Israel, performance was low of baseline and follow-up tests. In a multivariable analysis, the factors associated with higher adherence to most of the tests were male sex; older age; the presence of thyroid abnormalities, renal failure, and hypertension; and more frequent visits to primary care physicians.

Several previous studies (10–18) reported rates of performance of baseline and follow-up tests in conjunction with amiodarone treatment. These were often small-scale studies, with <200 patients (10–15). The largest was a UK study that included 1,413 individuals (16). Populations of previous studies from various countries [the United States (12–14), the United Kingdom (16), Taiwan (17), New Zealand (15), Saudi Arabia (10), and Israel (11)] were of similar age and sex distribution as the present study, with a mean age in the range of 50–78 years. Most of the studies reported male preponderance, in the range of 46%−71% (10–18). Our proportions of patients who performed baseline tests were within the ranges reported for thyroid function (43% vs. 41–97%) (10–15, 17, 18) and for liver function tests (49% vs. 44–97%) (10–15, 18). However, we report lower adherence to baseline chest X-rays: 21% vs. 56–84% (10–13, 15, 18) and to ECG, 15% vs. 58–100% (10–12, 15, 18). Regarding follow-up tests, we report a higher adherence rate for thyroid function tests, 70% vs. 9–64% (10, 11, 13, 15–18) and an adherence rate for liver function tests that was within the range of rates reported: 79% vs. 35–96% (10, 11, 13, 15, 16, 18).

The lower adherence to baseline tests such as ECG and chest X-rays could be attributed to poor physician knowledge or overload on the primary care physician, poor patient adherence, or poor documentation (see below in the discussion of the study limitations).

Liver and thyroid function, and pulmonary symptoms should be regularly monitored, to identify complications at an early stage. Amiodarone toxicity often presents atypically and insidiously, particularly in older patients. New symptoms in a patient taking amiodarone should always be considered as a potential adverse effect (1).

A multivariable analysis of factors associated with adherence to monitoring baseline and follow-up tests showed better adherence in younger patients and in males compared to baseline tests. Follow-up tests were also associated with some underlying conditions related to these tests. For example, thyroid abnormalities were associated with thyroid function test monitoring, while renal failure and hypertension were related to electrolyte monitoring. In addition, more frequent visits to primary care physicians were associated with a higher likelihood of completing these tests. A retrospective study of data from one hospital in Taiwan (17) identified a number of factors that associated with better monitoring of thyroid tests at baseline: follow-up by a cardiologist, a physician's experience, female sex, and younger age. In that study, the same factors, except for the patient's sex, were associated with regular follow-up of thyroid function.

Following their retrospective chart review of 100 patients in an Israeli hospital, Lavon and Goldman (11) suggested several solutions to the challenge of monitoring patients on amiodarone. These included automated alerts to physicians, automated referral letters to patients, creating a focused task force within the health providing organization, an awareness promotion campaign using various media channels, and the introduction of publicly reported quality measures for physicians and health organizations. Monitoring by clinical pharmacists was previously shown to increase the rate of monitoring for patients treated with amiodarone, both at baseline, and after 6 and 12 months of follow-up. Amiodarone-related adverse events were significantly fewer in the group monitored by pharmacists compared to the control group (17). Combining computerized alerts with collaboration between clinical pharmacists and physicians seems to be very effective. In a randomized controlled trial, the physicians and pharmacists in the intervention group teamed up to develop organization-specific guidelines for laboratory monitoring at the initiation of drug therapy. In collaboration with physicians, pharmacists were alerted to missing laboratory test results, ordered missing tests, reminded patients to obtain tests, assessed test completion, reviewed test results, and managed abnormal results. Amiodarone dispensing was monitored in 78.6% of the intervention group and in 51.4% of those who received usual care (*p* <0.001) (19).

The present study has several strengths. It is a population-based study of a large sample (more than 24,000 individuals), with a mean follow-up of 2.3 years, and including over 73,000 person-years. Data were extracted from the database of Clalit, which has demonstrated high reliability in numerous studies based on administrative data. The database has been described as having two decades of extensive identity-documentation-tagged, geo-coded, person-level detailed inpatient and outpatient clinical data. This clinical data repository offers a unique testing ground for new data-driven care models, while maintaining strict patient privacy and advanced cyber-security standards (20).

The study has a number of limitations. ECG tests may have been performed in the clinic but not documented in the designated field in the computerized records. Chest X-rays, ECG tests, and laboratory tests could have been performed in hospitals outside Clalit and not repeated, for example, during admissions to those hospitals. Physicians caring for patients who had normal blood tests more than 30 days prior to amiodarone treatment initiation might have thought that such data not need be repeated. As in other studies based on administrative data, standardization and harmonization in coding and data structure may introduce errors when linking and comparing data from various computerized sources. Clalit enrollees might not be representative of populations from other health provider organizations. As in other studies based on administrative databases, we did not have information on behavioral risk factors, disability, and functional status (21). When tests were not done, we had no information whether this happened because physicians failed to refer the patient, or because of poor patients' adherence. This information could direct efforts for improvement. Finally, variables potentially associated with adherence such as income and education level were not available.

In conclusion, adherence to monitoring of adverse effects of amiodarone therapy, especially prior to treatment initiation, was shown to be inadequate in a general Israeli population. Given that this research was carried out in a country with a high health care index score, similar results may be expected in other Western countries. This highlights the importance of enhancing education for patients and primary care physicians globally, on the importance of regular monitoring in patients treated with amiodarone.

## Data availability statement

The datasets presented in this article are not readily available because the study was conducted in the MDClone system and therefore data are unavailable for use by other researchers. Further inquiries should be directed to the corresponding author.

## Ethics statement

The studies involving humans were approved by Soroka Medical Center Ethics Committee. The studies were conducted in accordance with the local legislation and institutional requirements. Written informed consent for participation was not required from the participants or the participants' legal guardians/next of kin in accordance with the national legislation and institutional requirements.

## Author contributions

AF: Formal analysis, Writing – original draft, Writing – review & editing. RS: Formal analysis, Methodology, Software, Writing – original draft, Writing – review & editing. AS: Methodology, Supervision, Validation, Writing – original draft, Writing – review & editing. MM: Project administration, Writing – original draft, Writing – review & editing. OS-R: Methodology, Validation, Writing – original draft, Writing – review & editing. JD: Conceptualization, Data curation, Methodology, Supervision, Writing – original draft, Writing – review & editing.
